# A Novel Hybrid Parametric and Non-Parametric Optimisation Model for Average Technical Efficiency Assessment in Public Hospitals during and Post-COVID-19 Pandemic

**DOI:** 10.3390/bioengineering9010007

**Published:** 2021-12-27

**Authors:** Mirpouya Mirmozaffari, Reza Yazdani, Elham Shadkam, Seyed Mohammad Khalili, Leyla Sadat Tavassoli, Azam Boskabadi

**Affiliations:** 1 Department of Industrial Engineering, Dalhousie University, 5269 Morris Street, Halifax, NS B3H 4R2, Canada; 2 Department of Accounting, Technical and Vocational University (TVU), Tehran 1345120727, Iran; reza.yazdani@mail.com; 3 Department of Industrial Engineering, Faculty of Engineering, Khayyam University, Mashhad 9189747178, Iran; e.shadkam@khayyam.ac.ir (E.S.); m.khalili@khayyam.ac.ir (S.M.K.); 4 Department of Industrial Manufacturing and Systems Engineering, University of Texas at Arlington, Arlington, TX 76019, USA; leylasadat.tavassoli@mavs.uta.edu; 5 Department of Finance and Management Science, Carson College of Business, Washington State University, Pullman, WA 99163, USA; azam.boskabadi@wsu.edu

**Keywords:** COVID-19, artificial intelligence, data envelopment analysis, parametric and non-parametric models, public hospitals, average technical efficiency

## Abstract

The COVID-19 pandemic has had a significant impact on hospitals and healthcare systems around the world. The cost of business disruption combined with lingering COVID-19 costs has placed many public hospitals on a course to insolvency. To quickly return to financial stability, hospitals should implement efficiency measure. An average technical efficiency (ATE) model made up of data envelopment analysis (DEA) and stochastic frontier analysis (*SFA*) for assessing efficiency in public hospitals during and after the COVID-19 pandemic is offered. The DEA method is a non-parametric method that requires no information other than the input and output quantities. *SFA* is a parametric method that considers stochastic noise in data and allows statistical testing of hypotheses about production structure and degree of inefficiency. The rationale for using these two competing approaches is to balance each method’s strengths, weaknesses and introduce a novel integrated approach. To show the applicability and efficacy of the proposed hybrid VRS-CRS-SFA (*VCS*) model, a case study is presented.

## 1. Introduction

A disaster-resilient society relies heavily on healthcare infrastructure, such as hospitals, and it is important that these facilities stay operational at all times [1,2]. However, the COVID-19 pandemic serves as a strong reminder that we live in an ever-changing environment, and still built environments are vulnerable to disasters [3]. Effective responses to the pandemic have necessitated several deviations from traditional norms for health care delivery organisations. As a result of the COVID-19 pandemic, there have been several unique and severe financial loss concerns. There is an opportunity for health care executives to better prepare and alter their organisations for a future of unpredictability in the middle of these difficulties. Managing and controlling healthcare costs are always categorised among the challenging tasks for governments worldwide [4]. They also take care of delivering high-quality services and work efficiency simultaneously [5,6]. Therefore, decision-making and optimisation methods are widely used by decision-makers to cope with these and many other challenging conditions [7,8,9,10,11]. A plus point is that to evaluate the health sector’s effectiveness to support their healthcare units’ source utilisation, payers and purchasers may start using frontier productivity evaluation techniques. DEA and *SFA* are two of the most commonly used techniques for evaluating frontier productivity and efficiency, using quite distinct methodologies [12,13,14]. Notably, “non-statistical methods like DEA have some advantages and disadvantages. Assuming no statistical noise is among the drawbacks of this approach, being non-parametric and demanding limited conventions about the fundamental technology is advantageous. Alternatively, the disadvantage of *SFA* models is that they require strong assumptions about the form of the frontier but have the attraction of allowing for statistical noise” [15]. Unlikely, along with the *SFA* approach, various research types of the DEA analysis measure and evaluate the effectiveness of several sectors [16,17,18,19], which consists of the health sector [20,21,22,23]. Where the assumptions of neo-classical production theory are questionable and the evaluation error is unlikely to pose a significant threat, DEA is considered. *SFA*, on the other hand, should have the benefit of coping with significant measurement error and providing a near match to the properties of the underlying production technology. Gong and Sickles [24] demonstrate that “the more practical form becomes serious, the higher DEA’s demand (concerning *SFA*) becomes convincing”. The hospital industry and healthcare operations are exceptional examples of the application, where the amount of efficiency has proliferated over the past few years. Hospital units’ assessments have to date been carried out via DEA-based procedures. Nowadays, parametric, and non-parametric approaches have been applied to measure healthcare operations services’ efficiency performance analysis. The necessity for using competing techniques for frontier evaluation and effectiveness measurement has been emphasised by [25,26]. Therefore, the pair-wise comparison set is growing slowly once the newly emerged methods for the effectiveness evaluation appear, addressing and specifying the traditional approaches’ drawbacks. Thus, there is substantial interest in reconciling *SFA* and DEA in the efficiency analysis literature [27]. Finally, the following are some of the study’s major contributions.

This research assesses several efficiencies to provide insight into the hospital’s efficiency based on an innovative integrated or hybrid optimisation model over the first six months of the growing COVID-19 pandemic. This comparison is critical for public hospital practitioners who seek to analyse efficiency at the proper stage of its evolution.

i. Following the optimisation mentioned above, the statistical evaluation and comparison of three suggested models are applied, and the most efficient model is introduced. This statistical evaluation shows the positive and negative correlation between profit risk and efficiency.
ii. Considering multiple inputs and outputs based on the translog function, the VRS-CRS model is one of the current study’s novelties, which has not been studied in the previous research. The previous related papers merely consider CRS or VRS.
iii. Another novel aspect of the current study is the use of error-free unreplicated linear functional relationship (ULFR) to remove missing data and to present the least and the most efficient hospitals.
iv. The superior model and hospital are introduced after employing the novel combined optimisation approach. As a result, the findings of this study can assist decision-makers in eliminating irrelevant data and conducting more effective processes.

The following are the remaining sections of this study: Section 2 is a review of the literature. Section 3 presents the material and methods with seven parts of dataset description, research methodology, the non-parametric model, the parametric model, the proposed hybrid *VCS* model, the profit-risk evaluator (linear regression), and finally, the ULFR model. Section 4 provides the results and discussion. Section 5 contains the conclusion and future works on the practical implications.

## 2. Literature Review

Much effort has been devoted in recent years to develop and use approaches for improving hospital services [28]. Despite the significant potential for Operations Research (OR) to aid health decision-makers, as proven by multiple successful OR applications in other domains, there is a considerable research gap in utilising OR methodologies in assessing hospitals during emergency circumstances. Operation Research is a branch of study that use sophisticated analytical techniques to comprehend complicated systems and make appropriate decisions [29,30,31]. OR assists firms and organisations in a variety of methods, including simulation approaches [32,33,34], mathematical optimisation [35,36], and decision analysis [37,38]. OR, with its focus on boosting efficiency [39,40], cost-effectiveness [41,42], and decision making [43,44], is especially useful for analysing complex health sectors challenges.

DEA is one method that has been used widely by researchers in different research areas [45]. More recent studies [46,47,48,49,50] propose that *SFA* and DEA techniques, like other methods, can analyse the effect of dynamic environmental impacts on hospitals’ cost-efficiency. Additionally, Chirikos and Sear [51] for hospitals in the United States (US) and [15] as well as [52] for hospitals in the United Kingdom (UK) compared *SFA* and DEA, where both types of research found divergent estimations among the results from the two techniques. Finish hospitals’ cost-efficiency was examined by Linna [53] and realised that the results generated by *SFA* and DEA are similar. After that, [54,55,56,57] agreed with the previous scenario. The rest of the combined improved approaches, such as quantile regression or COLS, can seemingly have more credible estimates, which shows beneficial alternative methods in efficiency research. A plus point is that the researchers believe that utilising both DEA and *SFA* methods is necessary for various studies.

In a general view, scientists commonly utilised the DEA in the financial sector [58,59,60,61,62] and other sectors [20,63,64,65,66,67]. Moreover, in many studies, the researchers employed techniques to estimate hospital efficiencies, such as *SFA* and DEA. These frontier approaches primarily utilise an efficient approach to recognise the hospital sections’ effectiveness related to healthcare units’ reference set. *SFA*, as a parametric method hypothesising a practical form. Also, it utilises data to evaluate that function’s parameters econometrically using the whole decision-making units (DMUs) set. DEA is a non-parametric method utilising math programming for efficiency recognition. These two techniques do not have standard theoretical views [68,69]. On the other hand, DEA estimates the efficiency of a non-parametric measurement from the uncertain frontier [70]. According to Katharaki [5], both DEA and *SFA* approaches provide divergent efficiency estimates for numerous criteria such as statistical inputs and outputs definition, data availability, and noise. Nonetheless, variant modelling methods have both disadvantages and advantages. The selection of the most proper estimation technique must rely on the type of organisation type under examination, and the available data quality, as [71,72] indicated. The literature provides various suggestions regarding handling the environmental variable, as Jacobs et al. [73] noted. Katharaki [5] figured out researchers and the combination of techniques for measuring the efficiency and present environmental variables to make the decision-making properly. *SFA* required collecting the input variables depicted by total costs indicating that the cost efficiency is evaluated. In an ideal way, by increasing the patient health status, the health output should be examined. However, because this is not technically possible, intermediate outputs of various types are used as a replacement in almost all the hospital’s efficiency studies.

One of the most common *SFA* applications is the Cobb–Douglas functional or translog formula, typically applying one input or output, accompanied by current environmental aspects that are examined distinctly. The advantage of DEA is that compound production environments can be arranged with multiple inputs and outputs. *SFA* can distinguish among efficient units, but DEA has a limited ability to do this. Both techniques can distinguish among inefficient hospitals [52]. Regarding the paragraphs mentioned above and the research scope, the selection of multi-inputs and multi-outputs was adopted for the *SFA* translog formula, which has been applied in [74]. They applied these two approaches to evaluate the efficiency of the US 1471 hospitals and showed the Baumol effect once hospitals’ effectiveness declined with the trend of the labour costs soar gradually. As an example of other integrated methods, Keshtkar et al. [75] suggested a hybrid simulation technique that enables decision-makers to investigate the patient boarding problem. By combining ‘System Dynamic’ with ‘Discrete Event Simulation,’ the operator may address patient flow difficulties at both the macro and micro levels. The simulation is combined with ‘Design of Experiment (DOE)’ and ‘DEA’ to efficiently measure the administration’s operational influence. 

## 3. Materials and Methods

### 3.1. Dataset Description

**Figure 1:**
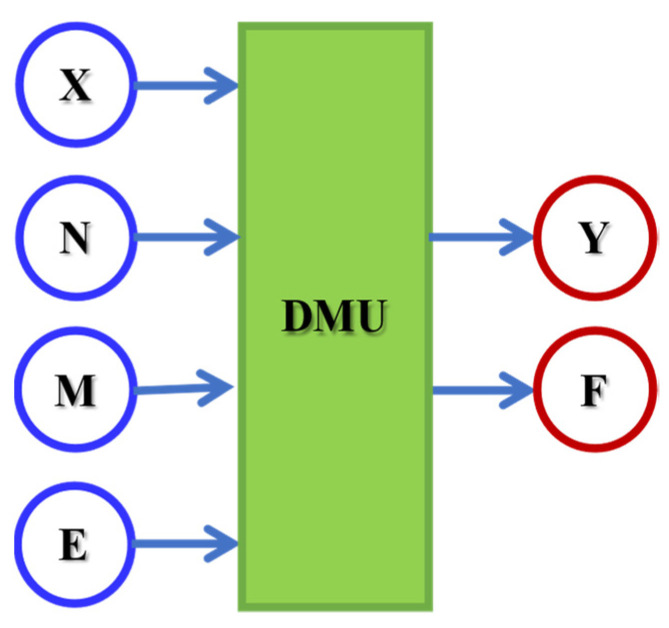
DEA model.

This research used a standard data set to assess hospital efficiency over a six-month period, from February to July 2020, corresponding to the first six months of the emerging COVID-19 pandemic. The data is derived from several sources, including the Middle East Ministry of Health (the Department of Health care). The information comes from 59 public hospitals and includes physician, personnel, and bed records. Similar to prior publications on assessing hospitals’ technical efficiency, the number of hospital beds as a proxy measure of capital is used. The number of corresponding full-time doctors and full-time matching nurses determines the inputs. In terms of outputs, hospitals are recognised to deliver a variety of services, necessitating a multi-output method. However, due to data availability, we limit our analysis to activities of inpatients and outpatients. Figure 1 depicts the general structure of the proposed model, as well as the input and output variables used to develop these studies. There are two output variables and four input variables. All variables are statistical variables.

Different completions of these variables have been used to develop various parts of the model and are described in the corresponding sections. Table 1 presents a descriptive analysis of the data.

### 3.2. Research Methodology

Hospital efficiency using a new hybrid framework that combines the non-parametric DEA model and the parametric *SFA* model in this study are assessed.

### 3.3. The Non-Parametric Model

Once an input vector${\vec{\;X}}_{0}$ and an output vector ${\vec{Y}}_{0}$ are generated in advanced manufacturing technology processes, the output $\lambda {\vec{Y}}_{0}$ can be generated from $\lambda {\vec{X}}_{0}$ when λ≥1. A set of production possibilities consisting of input-output pairs $\left(\vec{X,}\vec{Y}\right)$ is usually defined to guarantee that convexity and feasibility requirements are satisfied. In a *BCC*-*CCR* approach to this problem (see [71,72] among others), n DMUs are considered, and each of them is assumed to produce s outputs using m inputs. The following is the production possibility set:

(1) $$T_{BCC\text{-}CCR}=\left\{(\vec{X,}\vec{Y}):\vec{X}\geq \sum_{i=1}^{n}\lambda_{i}{\vec{X}}_{i}\wedge \vec{Y}\leq \sum_{i=1}^{n}\lambda_{i}{\vec{Y}}_{i}\wedge \sum_{i=1}^{n}\lambda_{i}\geq 1\wedge \;\lambda_{i}\geq 0\right\}$$

where $\left(\vec{X,}\vec{Y}\right)$ is a vector of dimension m+s with$\;\vec{X}={\left(x_{j}\right)}_{j=1,\ldots m}$ and $\vec{Y}={\left(y_{r}\right)}_{r=1,\ldots s}$. ${\vec{X}}_{i}={\left(x_{ji}\right)}_{j=1,\ldots m}$ is the input vector of the $i{\;}^{th}$ DMU, ${\vec{Y}}_{i}={\left(y_{ri}\right)}_{r=1,\ldots s}$ is the output vector of the $i{\;}^{th}\;$DMU, and $\lambda_{i}$ is the weight associated with the $i{\;}^{th}\;\mathrm{DMU}$. The inequalities describing the set are to be read component wise. Thus, in the input-oriented (${\mathrm{BCC}}_{\mathrm{IO}}{\text{-}\mathrm{CCR}}_{\mathrm{IO}}$) approach, the key aim is to obtain a virtual unit $DMU_{P}$ such that the input $\theta {\vec{X}}_{p}$ is less than or equal to ${\vec{X}}_{p}$ and the output is at least ${\vec{Y}}_{p}$. Therefore:

(2) Min θ

s.t.

(3) $$(\theta {\vec{X}}_{p},{\vec{Y}}_{p})\in T_{\mathrm{BBC}\text{-}\mathrm{CCR}}$$

Based on the definition of $\;T_{BCC\text{-}CCR}$ for a ${\mathrm{BCC}}_{\mathrm{IO}}{\text{-}\mathrm{CCR}}_{\mathrm{IO}}$ model, Equation (3) becomes [71,72]:

(4) Min θ

s.t.

(5) $$\sum_{i=1}^{n}\lambda_{i}x_{ji}\leq \theta x_{jp}\;\;\;\;\;\;\;\;\;\;\;\forall j=1,\ldots \ldots .,m$$

(6) $$\sum_{i=1}^{n}\lambda_{i}y_{ri}\geq y_{rp}\;\;\;\;\;\;\;\;\;\;\;\;\;\;\;\forall r=1,\ldots \ldots .,s$$

(7) $$\sum_{i=1}^{n}\lambda_{i}\geq 1\;\;\;\;\;\;\;\;\;\;\;\;\;\;\;\;\;\;\;\;\;\;\;\;\;\;\;\;\;\;\;\;\;\;\;\;$$

(8) $$\lambda_{i}\geq 0\;\;\;\;\;\;\;\;\;\;\;\;\;\;\;\;\;\;\;\;\;\;\;\;\;\;\;\;\;\;\;\;\;\;\;\;\;\;\forall i=1,\ldots ,n$$

Based on the statistical variables that we could defined using the data set, we formulated the following primal and dual ${\mathrm{BCC}}_{\mathrm{IO}}{\text{-}\mathrm{CCR}}_{\mathrm{IO}}$ models. The proposed primal ${\mathrm{BCC}}_{\mathrm{IO}}{\text{-}\mathrm{CCR}}_{\mathrm{IO}}$ model is as follows:

(9) $$Max\;\phi \;\sum_{r=1}^{s}Y_{ri}u_{r}+\sum_{z=1}^{q}F_{zi}a_{z}\;$$

s.t.

(10) $$\sum_{j=1}^{m}X_{ji}{}_{i}+\sum_{c=1}^{K}N_{ci}g_{c}+\sum_{h=1}^{d}M_{hi}l_{h}+\sum_{t=1}^{v}E_{ti}b_{t}\;\geq 1\;\;\;$$

(11) $$\sum_{r=1}^{s}Y_{ri}u_{r}+\sum_{z=1}^{q}F_{zi}a_{z}-\sum_{j=1}^{m}X_{ji}v_{i}-\sum_{c=1}^{K}N_{ci}g_{c}$$

(12) $$-\sum_{h=1}^{d}M_{hi}l_{h}-\sum_{t=1}^{v}E_{ti}b_{t}+w\;\leq 0\;\;\;\;\;\forall i=1,\ldots ,\;n$$

(13) s.t.

The proposed dual ${\mathrm{BCC}}_{\mathrm{IO}}{\text{-}\mathrm{CCR}}_{\mathrm{IO}}$ model is as follows:

(14) Min θ

s.t.

(15) $$\sum_{i=1}^{n}\lambda_{i}X_{ji}\leq \theta_{p}X_{jp}\;\;\;\;\;\forall j=1,\ldots ,m$$

(16) $$\sum_{i=1}^{n}\lambda_{i}N_{ci}\leq \theta_{p}N_{cp}\;\;\;\;\;\forall c=1,\ldots ,k$$

(17) $$\sum_{i=1}^{n}\lambda_{i}M_{hi}\leq \theta_{p}M_{hp}\;\;\;\;\;\forall h=1,\ldots ,\;d$$

(18) $$\sum_{i=1}^{n}\lambda_{i}E_{ti}\leq \theta_{p}E_{tp}\;\;\;\;\;\forall t=1,\ldots ,\;v$$

(19) $$\sum_{i=1}^{n}\lambda_{i}Y_{ri}\geq Y_{rp}\;\;\;\;\;\forall r=1,\ldots ,\;s$$

(20) $$\sum_{i=1}^{n}\lambda_{i}F_{zi}\geq F_{zp}\;\;\;\;\;\forall z=1,\ldots ,\;q$$

(21) $$\sum_{i=1}^{n}\lambda_{i}\geq 1$$

(22) $$\lambda_{i}\geq 0\;\;\;\;\;\;\;\;\;\forall i=1,\ldots ,\;n$$

(23) $$\theta_{p}\;free$$

The dimensionless parameters that appear in the primal $\;{\mathrm{BCC}}_{\mathrm{IO}}{\text{-}\mathrm{CCR}}_{\mathrm{IO}}$ model and in its dual are described in Table 2. Most of these parameters are completions of the input and output variables introduced in Figure 1 and Table 1. For the sake of completeness, the indices used to identify these completions are listed in Table 2 and Table 3 together with their meaning. The DMUs are the hospitals.

Lastly, DEA-SOLVER is used in this study to measure the technical efficiency scores of the proposed BCC_IO_-CCR_IO_ model.

### 3.4. The Parametric Model

The original formulation of the *SFA* model is based on the stochastic frontier production function and it can be implicitly expressed in matrix form as follow:

(24) $$O_{i}=I_{i}\beta +\varepsilon_{i},\;\forall i=1,\ldots ,n$$

where $I_{i}$ and $O_{i}$ are, respectively, the input and output vector of the $i^{th}$ *DMU* (i=1,…,n), β is the vector of unknown parameters to be assessed, and $\varepsilon_{i}$ is the composite error term. The error term is specified as the difference $\varepsilon_{i}=V_{i}+U_{i}$. The vector $V_{i}\;$is defined by random effect variables that account for the aggregate effects of unobserved factors on the production process. These factors are exogenous and cannot be controlled by the DMUs. The vector $U_{i}$ consists of non-negative random variables and is introduced to account for technical inefficiency in production. This inefficiency is commonly expressed in terms of output deviations from the frontier due to factors that can be controlled by the DMUs. Through the years, several alternative specifications of Equation (24) have been proposed due to the variety of research areas to which the model has been applied. Nevertheless, all these specifications can be considered as particular cases of the following more general matrix equation ([71,72]).

(25) $$O_{i}=F\left(I_{i};\beta \right)+\varepsilon_{i}$$

$F\left(I_{i};\beta \right)$ is a specified production function. When evaluating how efficiency evolves over time, Equation (25) becomes as follows ([71,72]):

(26) $$O_{it}=F\left(I_{it};\beta \right)+\varepsilon_{it}$$

where $I_{it}$ and $O_{it}$ are, respectively, the input and output vector of the $i^{th}$ *DMU* (i=1,…,n) for the period *t (*t=2,…,T*)*, $F\left(I_{it};\beta \right)$ is the production function, β is the vector of unknown parameters to be assessed, and $\varepsilon_{\mathrm{it}}=V_{\mathrm{it}}-{\;U}_{\mathrm{it}}$ is the composite error term.

When assessing hospitals’ technical efficiency, the $i^{th}$ *DMU* is identified with the $i^{th}$ hospital. The inputs and outputs of the $i^{th}$ hospital are completions of the variables introduced in the previous parts (Figure 1 and Table 1) at different periods. Thus, there are four inputs and two outputs for each hospital at each period. Moreover, due to the unbalance factors present at the different periods, the error term must be specified accordingly:

(27) $$\varepsilon_{\mathrm{it}}=V_{\mathrm{it}}-{\;U}_{\mathrm{it}}$$

$V_{\mathrm{it}}$ represents the statistical noise, that is, the effects of exogenous and uncontrollable factors that the hospitals cannot measure, such as measurement errors in the dependent variable, labour market conflicts, trade problems, access to raw material, quality, and left-out illustrative variables. $U_{it}$ denotes technical inefficiency, which is, the effects of those factors which can be monitored by the hospitals.

The value $U_{it}$ in the above Equation (27) is validated according to the following Equation (28), where $U_{i}$ represents the inefficiency level of the $i^{th}$ hospital for the period *t* and ρ is an unknown parameter.

(28) $$U_{it}=U_{i}e^{-\rho (t-T)}$$

Equation (29) shows how to evaluate the technical efficiency for the$\;i^{th}$ hospital at the $\;t^{th}$ time based on the suggested *SFA* model. This efficiency is denoted by $\;TE_{it}$.

(29) TEit=e−Ui e^−ρt−T= e−Uit  

Implementing a proper functional form for the production function of Equation (29) is a crucial task for completing the model assessment. Cobb-Douglas (CD) or Constant Elasticity of Substitution (CES) functions are often used in production function assessments. The CD and CES functions are both production functions that fulfil quasi-concavity and positive monotonicity. However, each of these functional forms place certain constraints on the parameters, such as exchange elasticity ([71,72]).

Recent research—a significant part of them regarding efficiency analysis of hospitals and implications of health information technology—has shown that the translog function can be a better option in assessing companies’ production and units’ efficiency. The translog function is a simplification of the CD function and represents a more flexible functional form since its definition includes second-order approximations. CD and translog functions are linear in terms of parameters, and their values are measured via least-squares techniques. However, the translog function has both linear and quadratic terms and has the advantage of being easier to use in the presence of multiple inputs and outputs, even if it is influenced by degrees of freedom and multicollinearity.

Thus, given the available data and the multiple inputs and the current study’s outputs settings, the translog function is employed. The translog form adopted for the production function in our study is provided by Equation (30).

(30) $$\begin{array}{cc} f\left(I_{it};\beta \right) & =\beta_{0}+\beta_{1}\ln\left(X_{it}\right)+\beta_{2}\ln\left(N_{it}\right)+\beta_{3}\ln\left(M_{it}\right)+\beta_{4}\ln\left(E_{it}\right)+\frac{1}{2}\beta_{5}\ln\left(X_{it}^{2}\right)\\ & +\frac{1}{2}\beta_{6}\ln\left(N_{it}^{2}\right)+\frac{1}{2}\beta_{7}\ln\left(M_{it}^{2}\right)+\frac{1}{2}\beta_{8}\ln\left(E_{it}^{2}\right)\\ & +\beta_{9}(\ln\left(X_{it}\right)\times \ln\left(N_{it}\right))\\ & +\beta_{10}(\ln\left(X_{it}\right)\times \ln\left(M_{it}\right))+\beta_{11}(\ln\left(X_{it}\right)\times \ln\left(E_{it}\right))\\ & +\beta_{12}(\ln\left(N_{it}\right)\times \ln\left(M_{it}\right))\\ & +\beta_{13}(\ln\left(N_{it}\right)\times \ln\left(E_{it}\right))+\beta_{14}(\ln\left(M_{it}\right)\times \ln\left(E_{it}\right)) \end{array}$$

where $X_{it}$ is the first input for the $i^{th}$ unit for the period t, $N_{it}$ is the second input for the $i^{th}$ unit for the period t, $M_{it}$ is the third input for the $i^{th}$ unit for the period t, $E_{it}$ is the fourth input for the $i^{th}$ unit for the period t, $\beta_{0}$ is the intercept or constant term, $\beta_{1}$, $\beta_{2},\;\beta_{3}\;\mathrm{and}\;\beta_{4}$ are the first-order results, $\beta_{5}$, $\beta_{6},\;\beta_{7}\;\mathrm{and}\;\beta_{8}$ are the second-order direct results, $\beta_{9}$, $\beta_{10},\;\beta_{11},\;\beta_{12},\beta_{13}$, $\beta_{14}$ are the second-order cross results. Using the translog form in Equation (30), the *SFA* model in Equation (26) becomes as follows.

(31) $$\begin{array}{cc} \ln\left(O_{it}\right) & =\beta_{0}+\beta_{1}\ln\left(X_{it}\right)+\beta_{2}\ln\left(N_{it}\right)+\beta_{3}\ln\left(M_{it}\right)+\beta_{4}\ln\left(E_{it}\right)+\frac{1}{2}\beta_{5}\ln\left(X_{it}^{2}\right)\\ & +\frac{1}{2}\beta_{6}\ln\left(N_{it}^{2}\right)+\frac{1}{2}\beta_{7}\ln\left(M_{it}^{2}\right)+\frac{1}{2}\beta_{8}\ln\left(E_{it}^{2}\right)\\ & +\beta_{9}(\ln\left(X_{it}\right)\times \ln\left(N_{it}\right))\\ & +\beta_{10}(\ln\left(X_{it}\right)\times \ln\left(M_{it}\right))+\beta_{11}(\ln\left(X_{it}\right)\times \ln\left(E_{it}\right))\\ & +\beta_{12}(\ln\left(N_{it}\right)\times \ln\left(M_{it}\right))\\ & +\beta_{13}(\ln\left(N_{it}\right)\times \ln\left(E_{it}\right))+\beta_{14}(\ln\left(M_{it}\right)\times \ln\left(E_{it}\right))+(V_{it}-U_{it}) \end{array}$$

The dimensionless parameters for the parametric model, Equation (31), are described in Table 4. As for the non-parametric case, most of these parameters are completions of the input and output variables introduced in Figure 1 and Table 1. 

### 3.5. The Proposed VRS-CRS-SFA (VCS) Model

The ATE model proposed in this study is a combination of the non-parametric *BCC*-*CCR* model and the parametric *SFA* model introduced in Section 3.3 and Section 3.4, respectively. Therefore, the proposed ATE model is a *VCS* model. Thus, in the following, we will refer to the proposed hybrid model as the *VCS* model. Based on Equation (32), for measuring the efficiency score of the newly proposed *VCS* model, the average efficiency score of the *BCC*-*CCR* and *SFA* models should be considered. That is:

(32) $$\begin{array}{c} Efficiency\;score\;of\;VCS=\\ =\frac{Efficiency\;score\;of\;BBC\text{-}CCR\;\mathrm{model}+Efficiency\;score\;of\;SFA\;}{2} \end{array}$$

### 3.6. Linear Regression Assessment or Profit-Risk Evaluator

Three linear regression forms were used to assess the positive or negative impacts of profit risk on the efficiency score of the *BCC*-*CCR*, *SFA*, and *VCS* models:

(33) $$EF{\left(BCC\text{-}CCR\right)}_{i}=\beta_{{\left(BCC\text{-}CCR\right)}_{0}}+\beta_{{\left(BCC\text{-}CCR\right)}_{i}}Apr_{{\left(BCC\text{-}CCR\right)}_{i}}+\varepsilon_{{\left(BCC\text{-}CCR\right)}_{i}}$$

(34) $$EF{\left(SFA\right)}_{i}=\beta_{{\left(SFA\right)}_{0}}+\beta_{{\left(SFA\right)}_{i}}Apr_{{\left(SFA\right)}_{i}}+\varepsilon_{{\left(SFA\right)}_{i}}$$

(35) $$EF{\left(VCS\right)}_{i}=\beta_{{\left(VCS\right)}_{0}}+\beta_{{\left(VCS\right)}_{i}}Apr_{{\left(VCS\right)}_{i}}+\varepsilon_{{\left(VCS\right)}_{i}}$$

Equations (33)–(35) represent the ATE scores of the $i^{th}$ hospital implementing the BBC-CCR, *SFA* and *VCS* models, respectively. $Apr_{{\left(BCC\text{-}CCR\right)}_{i}},\;Apr_{{\left(SFA\right)}_{i}},\;$and $Apr_{{\left(VCS\right)}_{i}}$ show the average profit risk of the $i^{th}$ hospital according to the *BCC*-*CCR*, *SFA*, and *VCS* models, correspondingly. $\beta_{{\left(BCC\text{-}CCR\right)}_{0}},\beta_{{\left(SFA\right)}_{0}},$ and $\beta_{{\left(VCS\right)}_{0}}$ are the intercept, the constant term, or the slope parameter of the *BCC*-*CCR*, *SFA*, and *VCS* models, respectively. $\beta_{{\left(BCC\text{-}CCR\right)}_{i}}$, $\beta_{{\left(SFA\right)}_{i}}$, and $\beta_{{\left(VCS\right)}_{i}}$ are the orderly derivatives of the *BCC*-*CCR*, *SFA* and *VCS* models, correspondingly, when considering the $i^{th}$ hospital. Finally, $\varepsilon_{{\left(BCC\text{-}CCR\right)}_{i}},\;\varepsilon_{{\left(SFA\right)}_{i}},\;$and $\varepsilon_{{\left(VCS\right)}_{i}}$ signify the error terms of the *BCC*-*CCR*, *SFA* and *VCS* models, respectively, when considering the $i^{th}$ hospital. 

### 3.7. ULFR Model

Assume that $APR_{{\left(BCC\text{-}CCR\right)}_{i}}$, $APR_{{\left(SFA\right)}_{i}},\;\mathrm{and}$ $APR_{{\left(VCS\right)}_{i}}$ are linearly related unobservable variables associated with $EF{\left(BCC\text{-}CCR\right)}_{i}$, $EF{\left(SFA\right)}_{i}$, and $EF{\left(VCS\right)}_{i}$, individually. Thus, the functional forms of the *BCC*-*CCR*, *SFA*, and *VCS* models are as follows, resepctively.

(36) $$\varphi_{{\left(BCC\text{-}CCR\right)}_{i}}=EF{\left(BCC\text{-}CCR\right)}_{i}=\beta_{{\left(BCC\text{-}CCR\right)}_{\alpha }}+\beta_{{\left(BCC\text{-}CCR\right)}_{f}}APR_{{\left(BCC\text{-}CCR\right)}_{i}}$$

(37) $$\varphi_{{\left(SFA\right)}_{i}}=EF{\left(SFA\right)}_{i}=\beta_{{\left(SFA\right)}_{\alpha }}+\beta_{{\left(SFA\right)}_{f}}APR_{{\left(SFA\right)}_{i}}$$

(38) $$\varphi_{{\left(VCS\right)}_{i}}=EF{\left(VCS\right)}_{i}=\beta_{{\left(VCS\right)}_{\alpha }}+\beta_{{\left(VCS\right)}_{f}}APR_{{\left(VCS\right)}_{i}}$$

where $\beta_{{\left(BCC\text{-}CCR\right)}_{\alpha }}$, $\beta_{{\left(SFA\right)}_{\alpha }}$, and $\beta_{{\left(VCS\right)}_{\alpha }}$ are $\beta_{{\left(BCC\text{-}CCR\right)}_{f}}$, $\beta_{{\left(SFA\right)}_{f}}$, and $\beta_{{\left(VCS\right)}_{f}}$. Moreover, the two equivalent random variables in the *BCC*-*CCR*, *SFA*, and *VCS* models are detected with errors $d_{{\left(BCC\text{-}CCR\right)}_{i}},\;d_{{\left(SFA\right)}_{i}},\;d_{{\left(VCS\right)}_{i}}$ and $e_{{\left(BCC\text{-}CCR\right)}_{i}},\;e_{{\left(SFA\right)}_{i}},\;e_{{\left(VCS\right)}_{i}}\;\left(i=1,2,\ldots .n\right)$, correspondingly. That is:

(39) $$\left\{\begin{array}{c} Ef{\left(BCC\text{-}CCR\right)}_{i}=\;EF{\left(BCC\text{-}CCR\right)}_{i}+d_{{\left(BCC\text{-}CCR\right)}_{i}}\\ Apr_{{\left(BCC\text{-}CCR\right)}_{i}}=APR_{{\left(BCC\text{-}CCR\right)}_{i}}+e_{{\left(BCC\text{-}CCR\right)}_{i}}\; \end{array}\right.$$

(40) $$\left\{\begin{array}{c} Ef{\left(SFA\right)}_{i}=EF{\left(SFA\right)}_{i}+d_{{\left(SFA\right)}_{i}}\\ Apr_{{\left(SFA\right)}_{i}}=APR_{{\left(SFA\right)}_{i}}+e_{{\left(SFA\right)}_{i}} \end{array}\right.$$

(41) $$\left\{\begin{array}{c} Ef{\left(VCS\right)}_{i}=\;EF{\left(VCS\right)}_{i}+d_{{\left(VCS\right)}_{i}}\\ Apr_{{\left(VCS\right)}_{i}}=APR_{{\left(VCS\right)}_{i}}+e_{{\left(VCS\right)}_{i}} \end{array}\right.$$

Equations (36)–(38) and Equations (39)–(41) represent the ULFR model in which *EF*(*BCC* − *CCR*)*_i_*, *EF*(*SFA*)*i*, *EF*(*VCS*)*i* and $APR_{{\left(BCC\text{-}CCR\right)}_{i}}$,$\;APR_{{\left(SFA\right)}_{i}},\;APR_{{\left(VCS\right)}_{i}}\;$are the only two variables of the *BCC*-*CCR*, *SFA*, and *VCS* models, respectively, and there is only one relation between$\;EF{\left(BCC\text{-}CCR\right)}_{i}\;$and $APR_{{\left(BCC\text{-}CCR\right)}_{i}}$, between$\;EF{\left(SFA\right)}_{i}$ and $APR_{{\left(SFA\right)}_{i}}$, and between$\;EF{\left(VCS\right)}_{i}$ and $APR_{{\left(VCS\right)}_{i}}$ Finally, *d*_(*BCC* − *CCR*)_*i*__, *d*_(*SFA*)_*i*__, *d*_(*VCS*)*_i_*_ and $e_{{\left(BCC\text{-}CCR\right)}_{i}},\;e_{{\left(SFA\right)}_{i},\;}e_{{\left(VCS\right)}_{i}}$ are random variables of the *BCC*-*CCR*, *SFA*, *VCS* models, respectively, which are mutually independent and normally distributed. To summarise our calculation, *VCS* proposed model is considered. *BCC*-*CCR* and *SFA* have the same evaluation, and they have not been considered in the following equations. The following conditions are considered for the *VCS* proposed model:

(42) $$E\left(d_{{\left(VCS\right)}_{i}}\;\right)=\;E(e_{{\left(VCS\right)}_{i}},\;\mathrm{Var}(d_{{\left(VCS\right)}_{i}})=\;\sigma_{d_{\left(VCS\right)}}^{2},\;\mathrm{Var}(e_{{\left(VCS\right)}_{i}})=\;\sigma_{e_{\left(VCS\right)}}^{2}\forall i$$

(43) $$Cov(d_{{\left(VCS\right)}_{i}},\;d_{{\left(VCS\right)}_{j}})=Cov(e_{{\left(VCS\right)}_{i}},\;e_{{\left(VCS\right)}_{j}})=0,\;i\neq j$$

(44) $$Cov(d_{{\left(VCS\right)}_{i}},\;e_{{\left(VCS\right)}_{j}})=0\;\forall i,j\;\;$$

The ratio of error variance in *VCS* proposed model is recognised as:

(45) $$\frac{\sigma_{e_{\left(VCS\right)}}^{2}}{\sigma_{d_{\left(VCS\right)}}^{2}}=\lambda$$

Consider the following 46, 47, 48, 49, and 50 assumptions in *VCS* proposed model before introducing the Equations (51)–(54):

(46) $$\bar{Ef\left(VCS\right)\;}=\frac{\sum Ef{\left(VCS\right)}_{i}}{n}$$

(47) $$\bar{Pr\left(VCS\right)\;}=\frac{\sum Pr{\left(VCS\right)}_{i}}{n}$$

(48) $$S_{yy}=\sum {\left(Ef{\left(VCS\right)}_{i}-\bar{Ef\left(VCS\right)\;}\right)}^{2}$$

(49) $$S_{xx}=\sum {\left(Pr{\left(VCS\right)}_{i}-\bar{Pr\left(VCS\right)\;}\right)}^{2}\;$$

(50) $$S_{xy}=\sum (Ef{\left(VCS\right)}_{i}-\bar{Ef\left(VCS\right)\;})\left(Pr{\left(VCS\right)}_{i}-\bar{Pr\left(VCS\right)\;}\right)$$

Based on the Equations (46)–(50) in the proposed *VCS* model, the maximum possibility evaluator of parameters in the *VCS* proposed model is introduced in Equations (51)–(53):

(51) $${\hat{\beta }}_{{\left(VCS\right)}_{\alpha }}=\bar{Ef\left(VCS\right)\;}-\beta_{{\left(VCS\right)}_{f}}\bar{Apr_{\left(VCS\right)}}$$

(52) $${\hat{\beta }}_{{\left(VCS\right)}_{f}\;}=\frac{\left(S_{yy}+\lambda S_{xx}\right)+{\left\{{\left(S_{yy}+\lambda S_{xx}\right)}^{2}+4\lambda S_{xy}^{2}\right\}}^{\frac{1}{2}}}{2S_{xy}}$$

(53) $$APR_{{\left(VCS\right)}_{i}}=\frac{\;\lambda Apr_{{\left(VCS\right)}_{i}}+{\hat{\beta }}_{{\left(VCS\right)}_{f}}\left(Ef{\left(VCS\right)}_{i}-{\hat{\beta }}_{{\left(VCS\right)}_{\alpha }}\right)}{\lambda +{\hat{\beta }}_{{\left(VCS\right)}_{f}}}$$

In the *VCS* suggested model, the sum of squared distances of the detected parts from the close-fitting line, or the residual sum of squares ($S_{E}$), is stated as Equation (54):

(54) $$\;SS_{E}=\frac{\sum {\left\{Ef{\left(VCS\right)}_{i}-\left({\hat{\beta }}_{{\left(VCS\right)}_{\alpha }}+{\hat{\beta }}_{{\left(VCS\right)}_{f}}Pr{\left(VCS\right)}_{i}\right)\right\}}^{2}}{\left(\lambda +{\hat{\beta }}_{{\left(VCS\right)}_{f}}{}^{2}\right)}\;=\frac{S_{yy}-2{\hat{\beta }}_{{\left(VCS\right)}_{f}}S_{xy}+{\hat{\beta }}_{{\left(VCS\right)}_{f}}{}^{2}S_{xx}}{\left(\lambda +{\hat{\beta }}_{{\left(VCS\right)}_{f}}{}^{2}\right)}$$

Consider that the error variance ratio in the suggested *VCS* model is one (λ=1). For special cases, in which λ≠1, it should be reduced with the case of λ=1 by dividing the detected amounts of $Ef\left(\mathrm{CAS}\right)$ by $\lambda^{\frac{1}{2}}$. As a result, Equation (55) is given:

(55) $$SS_{E}=\frac{S_{yy}-2{\hat{\beta }}_{{\left(VCS\right)}_{f}}S_{xy}+{\hat{\beta }}_{{\left(VCS\right)}_{f}}{}^{2}S_{xx}}{\left(1+{\hat{\beta }}_{{\left(VCS\right)}_{f}}{}^{2}\right)}$$

Consequently, the coefficient of determination for ULFR, considering the free value of in the proposed *VCS* model, is defined as Equation (56):

(56) $$R_{{\left(VCS\right)}_{f}}^{2}=\frac{SS_{R}}{S_{yy}}$$

In the *VCS* suggested model, $SS_{R}$ is the regression sum of squares and may be written as the following Equation (57):

(57) $$SS_{R}=S_{yy}-SS_{E}=S_{yy}-\frac{S_{yy}-2{\hat{\beta }}_{{\left(VCS\right)}_{f}}S_{xy}+{\hat{\beta }}_{{\left(VCS\right)}_{f}}{}^{2}S_{xx}}{\left(1+{\hat{\beta }}_{{\left(VCS\right)}_{f}}{}^{2}\right)}\;=\frac{{\hat{\beta }}_{{\left(VCS\right)}_{f}}{}^{2}S_{yy}+2{\hat{\beta }}_{{\left(VCS\right)}_{f}}S_{xy}-{\hat{\beta }}_{{\left(VCS\right)}_{f}}{}^{2}S_{xx}}{\left(1+{\hat{\beta }}_{{\left(VCS\right)}_{f}}{}^{2}\right)}$$

## 4. Results and Discussion

The technical efficiency of the three suggested models is assessed in the first step. We followed the advice of several reliable sources, including [76,77,78,79,80,81], when putting up our comparisons. This method is also applicable in various sectors, such as other healthcare management services as well as the energy sector [82,83,84].

### 4.1. Technical Efficiency Assessment Based on BCC-CCR, SFA, and VCS Models

The input-oriented approach dictates that a hospital can only be considered technically efficient if it can cut inputs while still delivering the expected outcomes. One is the efficiency score for staying on the best frontier line. In the first six months of the growing COVID-19 pandemic, the following models were evaluated for their technical efficiency: *BCC*-*CCR*, *SFA*, and novel *VCS* models.

**Figure 2:**
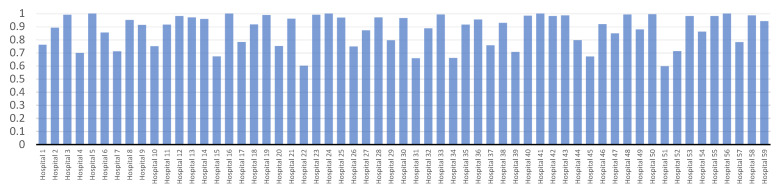
Hospitals’ technical efficiency results using *BCC*-*CCR*.

*BCC*-*CCR* model’s technical efficiency ratings for 59 hospitals are shown in Figure 2. In the first six months of COVID-19, the ATE score for hospitals was 0.876441. Hospitals have an inefficiency score of 12.3559% when it comes to the use of their current resources. During the first six months of pandemic COVID-19, hospitals No. 5, 16, 24, 41, and 56 with efficiency scores of one are efficient. while Hospitals No. 51 and 22 have the lowest efficiency scores of 0.598 and 0.602, respectively.

**Figure 3:**
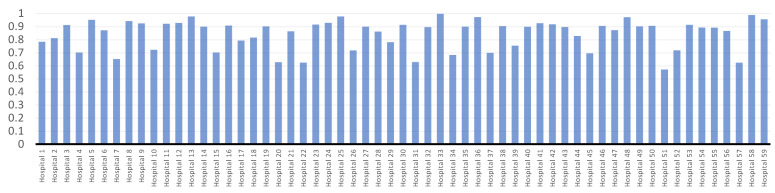
Hospitals’ technical efficiency results using *SFA*.

Figure 3 shows the *SFA* model’s technical efficiency ratings for 59 hospitals. The first six months of pandemic COVID-19 had an ATE score of 0.843475 across hospitals, according to the data. There is a 15.6525% inefficiency in hospitals’ use of their current resources, according to this data. The most efficient hospital during the first six months of the pandemic COVID-19 was hospital No. 58, with an efficiency score of 0.988. Hospitals No. 51, 57, and 22 have had the lowest efficiency scores of 0.571, 0.623, and 0.624, respectively.

**Figure 4:**
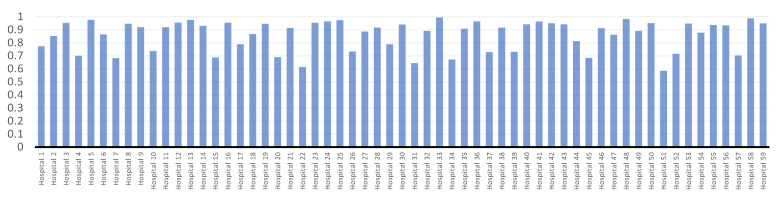
Hospitals’ technical efficiency results using *VCS*.

Technical efficiency ratings for 59 hospitals are shown in Figure 4. The first six months of the pandemic COVID-19 had an ATE score of 0.859958, according to statistics. This indicates that hospitals have a 14.0042% inefficiency using the available resources. With an efficiency score of 0.987, hospital No. 58 was the most effective throughout the first six months of the pandemic COVID-19. Hospitals No. 51, 22, and 31 had the lowest scores of 0.584, 6.13, and 0.643 respectively. 

As can be seen in Figure 2, Figure 3 and Figure 4, the first six months of the COVID-19 pandemic were the most inefficient at hospital No. 51 according to all recommended models. Using the available resources, it has an inefficiency score of more than 40% and efficiency scores of 0.598, 0.571, and 0.584 for *BCC*-*CCR*, *SFA*, and *VCS*.

### 4.2. ATE Evaluation for BCC-CCR, SFA, and VCS 

**Figure 5:**
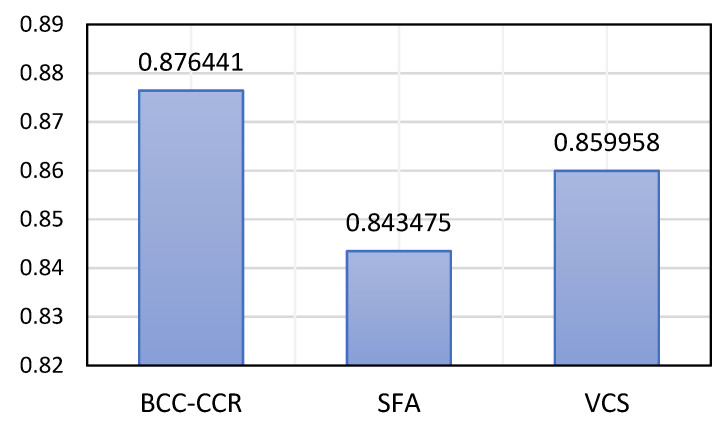
Evaluation of ATE for *BCC*-*CCR*, *SFA*, and *VCS*.

For 59 hospitals in the first six months of the COVID-19 pandemic, Figure 5 shows the ATE scores in the *BCC*-*CCR*, *SFA*, and *VCS* models. Based on the data in the sections above, the following relationship could be presented:

(58) $$ATE_{BCC\text{-}CCR}\left(0.876441\right)\geq ATE_{VCS}\left(0.859958\right)\geq ATE_{SFA}\left(0.843475\right)$$

Obviously, the ATE of the *BCC*-*CCR* ($ATE_{BCC\text{-}CCR}$) is higher than the ATE of the model *VCS* ($ATE_{VCS})$ and $ATE_{VCS}$ is higher than the ATE of *SFA* model ($ATE_{SFA}$). The *BCC*-*CCR* or DEA models cannot measure statistical noise, whereas in the *SFA* model, this is possible. In addition, the *SFA* allows DMUs to cross the efficiency frontier due to statistical noise and inefficiency scores. Finally, among the three mentioned models, the *BCC*-*CCR* model generates similar efficiency scores compared to the other two models.

### 4.3. Evaluation of Regression and ULFR

Table 5 summarises the results of statistical analyses performed using SPSS and SAS on the three models under consideration.

The results of the statistical evaluation with a significant 5% are presented in Table 5. As can be seen, a *p*-value of less than 5% with values of 0.0156 and 0.0021 for the proposed *BCC*-*CCR* and *VCS* models during the first six months of the COVID-19 pandemic, indicates a statistically significant relationship between profit risk and efficiency. Moreover, due to the low *p*-value, it can be said that profit risk has a positive effect on the financial performance of hospitals and provides better conditions for hospitals.

In addition, hospital managers face fewer challenges in wasting large amounts of profit in the first six months of the COVID-19 pandemic and can make more profit. Alternatively, a value of 0.1943 for *p*-value in the *SFA* model indicates a weak relationship between profit risk and *SFA* in this study (more than 0.05). Another result is the low-efficiency growth of 0.2971 ($\beta_{1}$) compared to the two values of 0.4783 and 0.5629 for the other two proposed models *BCC*-*CCR* and *VCS*, respectively. In fact, a 1% increase in the profit-risk of the *SFA* model creates a growth inefficiency of only 0.29%. While the efficiency growth of the proposed *VCS* model is twice the efficiency growth of the *SFA* model because the 1% increase in the profit-risk of the *VCS* model creates a performance of 0.56%. 

Finally, compared to the other two models, the proposed *VCS* model has a strong correlation between its coefficient and profit-risk because for the proposed *VCS* model the coefficient of determination for ULFR ($R_{f}^{2}$) and the coefficient of simple linear regression ($R^{2}$) is better and more than the other two models in the first six months of the COVID-19 pandemic. As a final point, these findings indicate that ULFR plays a more important role than linear regression because the coefficient of determination of ULFR ($R_{f}^{2}$) in the three proposed models (*BCC*-*CCR*, *SFA*, and *VCS*, with values 0.9965, 0.9941, 0.9998, respectively) are significantly more than (*BCC*-*CCR*, *SFA*, and *VCS*, with values 0.2283, 0.0825, and 0.2991, correspondingly).

### 4.4. VCS Assessment after ULFR Evaluation 

In this paper, fair comparisons have been followed in accordance with other computational methods [45,85,86,87,88]. The efficiency value of the proposed *VCS* model may have errors (due to some irrelevant data and missing values (that ULFR has been applied to eliminate. Table 6 shows the results for error-free efficiency of the proposed *VCS* model before and after the application of the ULFR model and the final ranking of hospitals after using the error-free ULFR method. Some hospitals have higher performance scores, and some hospitals have lower performance scores after implementing the error-free ULFR method.

Finally, hospital No. 58 is the most efficient hospital with an increase in efficiency from 0.987 to 0.999 and an increase in efficiency of 0.12%, and hospital No. 51 is the most inefficient hospital with a reduction in efficiency from 0.584 to 0.513 and a reduction efficiency of 0.71% in the first six months of the COVID-19 pandemic.

## 5. Conclusions and Future Works

In this paper, by combining *BCC*-*CCR* and *SFA* models, a hybrid method called *VCS* is presented and then this method is evaluated and compared with *SFA* and *BCC*-*CCR* methods in the first six months of the COVID-19 pandemic. The results show that the ATE score of VCSs is lower than the *BCC*-*CCR* model and the ATE score of *SFA* is lower than *VCS*. The average efficiency score of the nonparametric model *BCC*-*CCR* scores the highest, but unlike the parametric *SFA* model, they did not measure statistical noise. The most reliable approach has been used to evaluate and rank the models and hospitals and the positive or negative correlation between profit-risk and efficiency score has been investigated based on statistical analysis. Based on the ULFR coefficient and simple linear regression, the *VCS* model has the highest positive correlation between efficiency score and profit-risk compared to the two proposed models. In addition, to determine the most efficient and inefficient hospital and remove noisy and lost data, the ULFR method has been used and after applying this method, some hospitals received higher efficiency scores and others received lower efficiency scores. Hospital No. 58 and 51 received the highest and lowest scores, respectively. Simple linear regression coefficient and ULFR coefficient were the highest coefficients and based on the lowest *p*-value among the other proposed models, the proposed *VCS* model is the most appropriate method. It can be concluded that that many deficiencies such as the number of beds, staff, doctors have affected patient care during the pandemic. Hospitals also raised concerns that the pandemic has aggravated current gaps regarding care and health consequences. In future research, the combination of other parametric methods such as the thick frontier approach (TFA) and deterministic frontier approach (DFA) and well-known non-parametric methods such as *CCR*-*BCC* in the DEA can be considered. Furthermore, the verified simulation model can be run to prove the validity and efficiency of the mentioned approaches as a potential robustness test.

## Figures and Tables

**Table 1:** Statistical analyse of dataset.

Stat: February–July 2020	Description	Mean	SD
X	The total number of physicians	449	131
N	The total number of other personnel	1062	300
M	The total number of beds	574	168
E	The total operating costs	62,549.651	29,769.91
Y	The total number of inpatient admissions	7144	35,799
F	The total number of outpatient visits	49,574	175,240

**Table 2:** Explanation of the parameters for primal and dual ${\mathrm{BCC}}_{\mathrm{IO}}\text{-}{\mathrm{CCR}}_{\mathrm{IO}}$.

Dimensionless Parameter	Description
$\lambda_{i}$	Non-negative individual value (dual variables categorise the benchmarks for inefficient parts) for the *i^th^* *DMU*
$X_{ji}$	*j^th^* completion of the input variable X (total number of physicians) for the *i^th^* *DMU*
$N_{ci}$	*c^th^* completion of the input variable N (total number of other personnel) for the *i^th^* *DMU*
$M_{hi}$	*h^th^* completion of the input variable *M* (total number of beds) for the *i^th^* *DMU*
$E_{ti}$	*t^th^* completion of the input variable E (total of operating costs) for the *i^th^* *DMU*
$Y_{ri}$	*r^th^* completion of the output variable ^Y^ (total number of inpatient admissions) for the *i^th^* *DMU*
$F_{zi}$	*z^th^* completion of the output variable F (total number of outpatient visits) for the *i^th^* *DMU*
w	Free of sign individual value for variable return to scale
$v_{j}$	The weight designated to input *X_ji_*
$g_{c}$	The weight designated to input *N_ci_*
$l_{h}$	The weight designated to input *M_hi_*
$b_{t}$	The weight designated to input *E_ti_*
$u_{r}$	The weight designated to output *Y_ri_*
$a_{z}$	The weight designated to output *F_zi_*
ϕ	Individual value and real primal-variable demonstrating the value of efficiency
θ	Individual value and real dual-variable demonstrating the value of efficiency
$\theta_{p}$	Free of sign dual individual value for the fixed *p^th^* *DMU*
$X_{jp}$	*j^th^* completion of the dual input variable X (total number of physicians) for the fixed *p^th^* *DMU*
$N_{cp}$	*c^th^* completion of the dual input variable N (total number of other personnel) for the fixed *p^th^* *DMU*
$M_{hp}$	*h^th^* completion of the dual input variable M (total number of beds) for the fixed *p^th^* *DMU*
$E_{tp}$	*t^th^* completion of the dual input variable E (total of operating costs) for the fixed *p^th^* *DMU*
$Y_{rp}$	*r^th^* completion of the dual output variable Y (total number of inpatient admissions) for the fixed *p^th^* *DMU*
$F_{zp}$	*z^th^* completion of the dual output variable F (total number of outpatient visits) for the fixed *p^th^* *DMU*

**Table 3:** Explanation of the parameters for primal and dual ${\mathrm{BCC}}_{\mathrm{IO}}\text{-}{\mathrm{CCR}}_{\mathrm{IO}}$.

Index	Description
*n*	Total number of DMUs
*m*	Total number of completions observed for the input variable X (total number of physicians)
*k*	Total number of completions observed for the input variable *N* (total number of other personnel)
*d*	Total number of completions observed for the input variable *M* (total number of beds)
*v*	Total number of completions observed for the input variable *E* (total of operating costs)
*s*	Total number of completions observed for the output variable *Y* (total number of inpatient admissions)
*q*	Total number of completions observed for the output variable *F* (total number of outpatient visits)
*i*	Index of the generic *DMU*, *DMU*_*i*_; *i* = 1, …, *n*
*p*	Index of the fixed *p^th^* *DMU*, *DMU*_*p*_
*j*	Index of a completion observed for the input variable *X*; *j* = 1, …, *m*
*c*	Index of a completion observed for the input variable *N*; *c* = 1, …, *k*
*h*	Index of a completion observed for the input variable *M*; *h* = 1, …, *d*
*t*	Index of a completion observed for the input variable *E*; *t* = 1, …, *v*
*r*	Index of a completion observed for the output variable *Y*; *r* = 1, …, *s*
*z*	Index of a completion observed for the output variable *F*; *z* = 1, …, *q*

**Table 4:** Explanation of the parameters for primal and dual *SFA*.

Dimensionless Parameter	Description
$X_{it}$	$\mathrm{Total}\;\mathrm{number}\;\mathrm{of}\;\mathrm{physicians}\;(\mathrm{first}\;\mathrm{input})\;\mathrm{for}\;\mathrm{the}\;i^{th}$ hospital for the period t
$N_{it}$	$\mathrm{Total}\;\mathrm{number}\;\mathrm{of}\;\mathrm{other}\;\mathrm{personnel}\;(\sec\mathrm{ond}\;\mathrm{input})\;\mathrm{for}\;\mathrm{the}\;i^{th}\;$hospital for the period t
$M_{it}$	$\mathrm{Total}\;\mathrm{number}\;\mathrm{of}\;\mathrm{beds}\;(\mathrm{third}\;\mathrm{input})\;\mathrm{for}\;\mathrm{the}\;i^{th}$ hospital for the period t
$E_{it}$	$\mathrm{Total}\;\mathrm{operating}\;\cos\mathrm{ts}\;(\mathrm{fourth}\;\mathrm{input})\;\mathrm{for}\;\mathrm{the}\;i^{th}$ hospital for the period t
$O_{it}$	$\mathrm{Output}\;\mathrm{vector}\;(\mathrm{total}\;\mathrm{number}\;\mathrm{of}\;\mathrm{inpatient}\;\mathrm{admissions}\;\mathrm{for}\;\mathrm{the}\;\mathrm{first}\;\mathrm{output}\;\mathrm{and}\;\mathrm{total}\;\mathrm{number}\;\mathrm{of}\;\mathrm{outpatient}\;\mathrm{visits}\;\mathrm{for}\;\mathrm{the}\;\sec\mathrm{ond}\;\mathrm{output})\;\mathrm{for}\;\mathrm{the}\;i^{th}$ hospital for the period t
$V_{it}$	$\mathrm{Random}\;\mathrm{error}\;\mathrm{for}\;\mathrm{the}\;i^{th}$ hospital for the period t
$U_{it}$	Non-negative random variable (or technical inefficiency) for the $i^{th}$ hospital for the period t
$\ln\left(X_{it}\right)$	$\mathrm{Inverse}\;\mathrm{of}\;\mathrm{natural}\;\mathrm{exponent}\;\mathrm{of}\;\mathrm{total}\;\mathrm{number}\;\mathrm{of}\;\mathrm{physicians}\;\mathrm{for}\;\mathrm{the}\;i^{th}$ hospital for the period t
$\ln\left(N_{it}\right)$	$\mathrm{Inverse}\;\mathrm{of}\;\mathrm{natural}\;\mathrm{exponent}\;\mathrm{of}\;\mathrm{total}\;\mathrm{number}\;\mathrm{of}\;\mathrm{other}\;\mathrm{personnel}\;\mathrm{for}\;\mathrm{the}\;i^{th}\;$hospital for the period t
$\ln\left(M_{it}\right)$	$\mathrm{Inverse}\;\mathrm{of}\;\mathrm{natural}\;\mathrm{exponent}\;\mathrm{of}\;\mathrm{total}\;\mathrm{number}\;\mathrm{of}\;\mathrm{beds}\;\mathrm{for}\;\mathrm{the}\;i^{th}$ hospital for the period t
$\ln\left(E_{it}\right)$	$\mathrm{Inverse}\;\mathrm{of}\;\mathrm{natural}\;\mathrm{exponent}\;\mathrm{of}\;\mathrm{total}\;\mathrm{operating}\;\cos\mathrm{ts}\;\mathrm{for}\;\mathrm{the}\;i^{th}$ hospital for the period t
$\ln\left(Y_{it}\right)$	$\mathrm{Inverse}\;\mathrm{of}\;\mathrm{natural}\;\mathrm{exponent}\;\mathrm{of}\;\mathrm{the}\;\mathrm{output}\;\mathrm{variable}\;(\mathrm{total}\;\mathrm{number}\;\mathrm{of}\;\mathrm{inpatient}\;\mathrm{admissions}\;\mathrm{for}\;\mathrm{the}\;\mathrm{first}\;\mathrm{output}\;\mathrm{and}\;\mathrm{total}\;\mathrm{number}\;\mathrm{of}\;\mathrm{outpatient}\;\mathrm{visits}\;\mathrm{for}\;\mathrm{the}\;\sec\mathrm{ond}\;\mathrm{output})\;\mathrm{for}\;\mathrm{the}\;i^{th}$ hospital for the period t
$\beta_{0}$	Intercept or constant term
$\beta_{1}$	First-order result of the inverse of natural exponent for the first input *X_it_*
$\beta_{2}$	First-order result of the inverse of natural exponent for the second input *N_it_*
$\beta_{3}$	First-order result of the inverse of natural exponent for the third input *M_it_*
$\beta_{4}$	First-order result of the inverse of natural exponent for the fourth input *E_it_*
$\beta_{5}$	Second-order direct result of the inverse of natural exponent for the first input *X_it_*
$\beta_{6}$	Second-order direct result of the inverse of natural exponent for the second input *N_it_*
$\beta_{7}$	Second-order direct result of the inverse of natural exponent for the third input *M_it_*
$\beta_{8}$	Second-order direct result of the inverse of natural exponent for the fourth input *E_it_*
$\beta_{9}$	Second-order cross result of the product of the inverse of natural exponents of the first and second inputs for the $i^{th}$ hospital for the period t
$\beta_{10}$	Second-order cross result of the product of the inverse of natural exponents of the first and third inputs for the $i^{th}$ hospital for the period t
$\beta_{11}$	Second-order cross result of the product of the inverse of natural exponents of the first and fourth inputs for the $i^{th}$ hospital for the period t
$\beta_{12}$	Second-order cross result of the product of the inverse of natural exponents of the second and third inputs for the $i^{th}$ hospital for the period t
$\beta_{13}$	Second-order cross result of the product of the inverse of natural exponents of the second and fourth inputs for the $i^{th}$ hospital for the period t
$\beta_{14}$	Second-order cross result of the product of the inverse of natural exponents of the third and fourth inputs for the $i^{th}$ hospital for the period t

**Table 5:** Statistical assessment of the *BCC*-*CCR*, *SFA*, and *VCS*.

Model	Coefficients ($\beta_{1}$)	Coefficient Determination of Simple Linear Regression ($R^{2}$)	Coefficient Determination of ULFR ($R_{f}^{2}$)	*p*-Value
*BCC*-*CCR*	0.4783	0.2283	0.9965	0.0156
*SFA*	0.2971	0.0825	0.9941	0.1943
*VCS*	0.5629	0.2991	0.9998	0.0021

**Table 6:** Efficiency assessment of the hospitals for proposed hybrid *VCS* model before using ULFR, after using ULFR and final ranking after applying ULFR.

Hospitals	Before ULFR	After ULFR	Ranking	Hospitals	Before ULFR	After ULFR	Ranking
1	0.772	0.774	43	31	0.643	0.658	56
3	0.951	0.965	14	33	0.995	0.998	2
4	0.700	0.702	48	34	0.671	0.682	54
5	0.975	0.982	8	35	0.907	0.914	28
6	0.863	0.852	38	36	0.963	0.980	9
7	0.681	0.692	53	37	0.728	0.694	52
8	0.946	0.941	20	38	0.916	0.933	22
9	0.919	0.911	29	39	0.730	0.712	45
10	0.736	0.700	49	40	0.941	0.979	10
11	0.918	0.924	26	41	0.962	0.955	17
12	0.954	0.943	19	42	0.949	0.991	4
13	0.974	0.983	7	43	0.941	0.904	30
14	0.929	0.936	21	44	0.812	0.842	37
15	0.687	0.665	55	45	0.683	0.699	50
16	0.953	0.967	13	46	0.911	0.895	33
17	0.787	0.771	44	47	0.860	0.813	39
18	0.866	0.878	35	48	0.982	0.995	3
19	0.945	0.964	15	49	0.890	0.888	34
20	0.689	0.641	57	50	0.950	0.928	24
21	0.912	0.902	31	51	0.584	0.513	59
22	0.613	0.599	58	52	0.715	0.697	51
23	0.953	0.987	5	53	0.947	0.962	16
24	0.964	0.985	6	54	0.877	0.845	41
25	0.973	0.977	11	55	0.936	0.972	12
26	0.733	0.705	47	56	0.933	0.900	32
27	0.885	0.873	36	57	0.702	0.710	46
28	0.916	0.931	23	58	0.987	0.999	1
29	0.788	0.799	42	59	0.948	0.947	18
30	0.939	0.926	25				

## Data Availability

The data used in the study is available with the authors and can be shared upon reasonable requests.
